# Pig Health Assessment Framework Based on Behavioural Analysis

**DOI:** 10.3390/ani15243650

**Published:** 2025-12-18

**Authors:** Shuqin Tu, Boyang Tan, Aqing Yang, Hairan Yang, Yizhi Luo, Yuan Fang, Zicong Xu

**Affiliations:** 1 College of Mathematics and Informatics, South China Agricultural University, Guangzhou 510642, China; 2 College of Computer Science, Guangdong Polytechnic Normal University, Guangzhou 510665, China; 3 Institute of Facility Agriculture, Guangdong Academy of Agricultural Sciences, Guangzhou 510640, China; 4 College of Computer Science and Electronic Engineering, Hunan University, Changsha 410082, China

**Keywords:** pig, multi-object tracking, health assessment, ByteTrack

## Abstract

The long-term behavioural analysis and health assessment of Pigs are essential for intelligent management in modern pig farming. Manual tracking and behaviour analysis for constructing health assessment systems are often subjective, inconsistent, and lack sufficient accuracy. To overcome these challenges, this study proposes a health assessment framework for pigs based on multi-object behaviour tracking and analysis under large-scale pig farming. The proposed framework consists of three modules: an improved ByteTrack-based multi-object tracking (MOT) module, a behaviour statistics and analysis module, and a health assessment module. The pipeline involves using the MOT module to obtain pigs’ behavioural data, followed by the behaviour analysis module and health assessment module to analyse and evaluate the health status of the pigs. Two datasets comprising 18 videos of healthy pigs and 10 videos of unhealthy pigs were created to validate the framework. Experimental results demonstrated that the improved ByteTrack algorithm achieved high performance in MOT metrics, including a High-Order Tracking Accuracy (HOTA) of 74.0%, Multiple Object Tracking Accuracy (MOTA) of 92.2%, Identification F1 Score (IDF1) of 89.4%, and 43 identity switches (IDs). The behaviour statistics derived from these tracking results enabled reliable inputs for the health assessment model, which accurately assesses the health status of each pig. The results demonstrate that the proposed framework provides an effective solution and reliable technical support for pig health monitoring in modern pig farming.

## Simple Summary

This study proposes a pig health assessment framework based on multi-object tracking (MOT), which automatically tracks and quantifies pig behaviour in large-scale pig farming. The framework consists of an MOT module, a behaviour statistics and analysis module, and a health assessment module. Through the MOT module and the behaviour statistics and analysis module, the framework accurately captures the behaviour patterns of individual pigs, which are then used by the health assessment module to evaluate their health status. Experimental results show that the framework performs effectively in both tracking accuracy and health assessment, providing reliable technical support for health monitoring in pig farming.

## 1. Introduction

Daily activity levels of pigs serve as a key indicator for analysing their health status. Pig daily behaviours exhibit regular fluctuations, and irregular changes in behaviour often signal problems such as disease, improper nutrition, or environmental stress [1]. However, in pig farming operations, disease prevention and control still rely mainly on manual monitoring of health status changes, which is time-consuming, labour-intensive, subjective, inefficient, and costly [2]. For major swine diseases such as African swine fever (ASF), relying solely on the manual recognition of mild clinical symptoms for passive detection often leads to a delay of several weeks before the pathogen can be identified in large-scale pig farms, allowing the pathogen to spread and cause substantial losses. Several studies have shown that the direct and indirect economic costs of a single outbreak or ongoing epidemic can range from tens of millions to several billions of USD, depending on the country, region, and specific disease [3,4,5,6,7]. Early detection and real-time monitoring systems can significantly reduce these losses. With the continuous advancement of deep learning technology, it is now possible to non-invasively and with minimal stress employ computer vision methods to efficiently and accurately collect each pig’s daily behavioural data. This enables real-time monitoring of both behaviour and the health status of group-housed pigs, which plays a crucial role in managing herd health in pig farms.

In recent years, with the rapid development of computer vision technology, multi-object tracking (MOT) methods have been applied to livestock identification and tracking [8,9,10,11,12,13]. For example, Huang et al. proposed an improved model combining YOLOv5 and DeepSORT for pig tracking and counting, which achieved a Multiple Object Tracking Accuracy (MOTA) of 85.32% [8]. To address the issues of missed and false detections caused by complex environments in cattle detection and tracking, Zheng et al. introduced an enhanced MOT method named YOLO-BYTE. Compared to the original algorithm, their method improved the High-Order Tracking Accuracy (HOTA) by 4.4%, the Multiple Object Tracking Accuracy (MOTA) by 6.1%, the Identification F1 Score (IDF1) by 3.8%, and reduced identity switches (IDs) by 37.5% [9]. Cao et al. developed a model integrating an improved YOLOv5x with DeepSORT for tracking and counting sheep. Experimental results indicated a test accuracy of 97.10%, with a dynamic counting error rate of 5% [10]. Wu et al. proposed a model combining YOLOv5x with an enhanced DeepSORT algorithm to track and count four species of migratory fish. The model achieved an average counting accuracy of 75.5% across the four species [11]. Tu et al. introduced an MOT method for pigs called RpTrack, which effectively addresses challenges such as uneven lighting and irregular pig movements [12]. Li et al. proposed a method, DYTB, for multi-object pig tracking, achieving a pig detection accuracy of 98.3%, along with tracking accuracies of 95.3% and 97.1% [13]. The above works have demonstrated the feasibility of individual tracking in group-housed environments. However, these methods focus solely on identification and tracking, lacking deeper investigation into areas such as applying MOT technology for detailed behavioural analysis and further developing automated health assessment frameworks based on these behavioural patterns.

Moreover, in the field of pig target tracking, many studies have further explored the recognition and tracking of pig behaviours [14,15,16,17,18]. For example, Alameer et al. proposed a feeding behaviour recognition method based on grayscale video frames and an enhanced GoogleNet (Inception V3) architecture, achieving a high accuracy of 99.4% in identifying feeding-related behaviours [14]. Huang et al. introduced HE-YOLO for the real-time recognition of postures and behaviours in group-housed pigs. The model achieved a mean average precision (mAP) ranging from 94.43% to 99.25% across four posture categories [15]. Odo et al. developed a deep learning-based video analysis approach for the automatic quantification of pig ear-biting behaviour. DeepSORT and centroid tracking algorithms were used to quantify the behaviour [16]. Taiwo et al. proposed a method for dynamically recognising aggressive behaviours in pigs using a Vision Transformer, which achieved an accuracy of 82.8% and an F1 score of 82.7% [17]. Luo et al. presented a lightweight multi-behaviour recognition algorithm (PBR-YOLO) for piglets. It achieved an accuracy of 82.7% and an mAP of 78.5% [18]. However, many of these studies have remained focused on behaviour recognition and tracking, without further extending these techniques to health assessment applications. This presents a valuable opportunity for future research to integrate behaviour-based insights into intelligent animal health monitoring systems.

As a further extension of pig behaviour recognition and tracking, current research on pig health assessment predominantly relies on wearable sensors such as Bluetooth Low-Energy (BLE) devices and Radio-Frequency Identification (RFID) systems to record behavioural data and monitor health status [19,20,21,22]. For instance, de Bruijn et al. used RFID to collect feeding and drinking behaviour data and developed a model for the early detection of potential health issues in pigs [19]. Lee et al. proposed a pig monitoring and identification system that integrates BLE tags with WBLCX antennas to support individual recognition and counting in real-time [20]. Huang et al. introduced an analytical method based on electronic ear tag data, employing machine learning techniques to identify differences in activity levels and temperature changes between healthy and unhealthy pigs [21]. Similarly, Yin et al. proposed a method combining statistical analysis and machine learning to differentiate activity levels between healthy and unhealthy pigs [22]. Their approach demonstrated the feasibility of using hourly activity data to predict health problems, providing valuable insights for pig health assessments. However, wearable sensor-based solutions in large-scale pig farming face several limitations, including the risk of device detachment, high equipment costs, and difficulties in maintaining reliable monitoring accuracy.

In contrast, using pig behaviour tracking for health assessments in large-scale pig farms offers a cost-effective and device-free alternative to wearable sensors [23,24]. These approaches effectively reduce equipment costs and eliminate issues related to device detachment. For example, Bhujel et al. proposed a computer vision-based automatic detection approach to investigate the effects of varying greenhouse gas concentrations on pig health [23]. Xu et al. introduced an automatic evaluation method that combines the YOLOv5s model with XGBoost to quantify the activity level of group-housed pigs. Their findings suggest that the collective activity level of pigs serves as an indirect indicator of health status, enabling health assessment through motion quantification [24]. Despite these promising advances, few studies have leveraged MOT results to further analyse and assess the health status of pigs. This highlights a critical research gap and presents opportunities for integrating long-term behaviour tracking results with intelligent health monitoring systems for health assessment application in precision livestock farming.

To address the challenges, this study proposes a pig health assessment framework based on multi-object behaviour tracking and analysis results. The framework contains three modules: a multi-object tracking (MOT) module, a behaviour statistics and analysis module, and a health assessment module. The MOT module employs an improved ByteTrack to track the ‘lie’, ‘stand’, ‘eat’, and ‘other’ behaviour in pigs. The behaviour statistics and analysis module analyses the long-term tracking data to calculate the time proportion that each pig spends on the identified behaviour. The health assessment module assesses the health status of pigs based on the results of the behavioural statistics and analysis module and expert guidance.

The main contributions of this study are summarised as follows.

(1) We propose a pig health assessment framework to analyse and evaluate the health status of individual pigs in an intelligent monitoring environment.
(2) An improved ByteTrack algorithm is employed to enhance behaviour recognition and MOT performance in large-scale pig farming.
(3) Based on the pig ID and behaviour categories information from MOT, we develop a behaviour statistics and analysis module to complete long-term (24 h) behavioural analysis for pigs.
(4) To assess the health status of group-housed pigs, we implement a health assessment module and validate it in two video datasets including both healthy and unhealthy pigs.

## 2. Material and Methods

### 2.1. Materials

#### 2.1.1. Video’s Datasets of Healthy and Unhealthy Pigs

**Figure 1:**
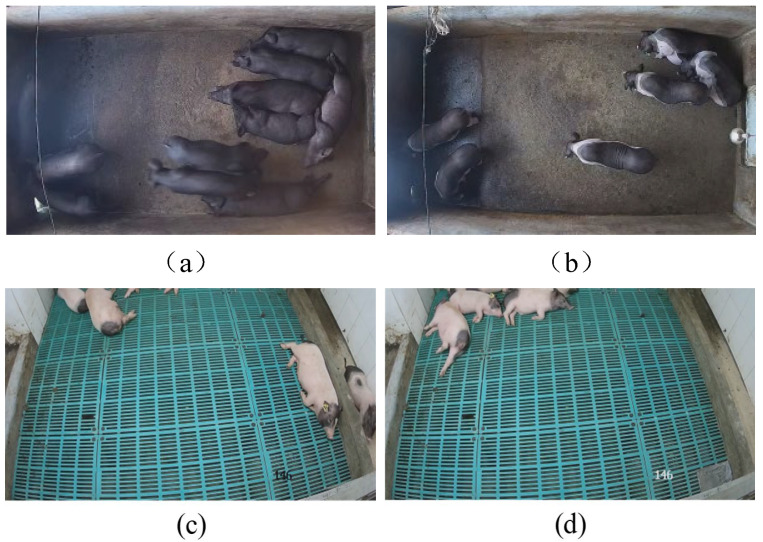
The examples from the datasets. (**a**,**b**) Examples of healthy pigs. (**c**,**d**) Examples of unhealthy pigs.

The health pig dataset was collected through continuous 24 h monitoring from 8 April to 8 May 2023, at the Lejiazhuang Breeding Base in Sanshui District, Foshan City, Guangdong Province, China. A surveillance camera was centrally mounted above the breeding area, capturing the entire region from a downward angle. The camera operated at a sampling rate of 30 frames per second (30 fps) with an image resolution of 1540 × 930 pixels. The dataset included two pig breeds: 60-day-old black pigs and 90-day-old spotted pigs belonging to the breed officially named ‘MeiHua’. Examples of healthy pigs are shown in Figure 1a,b.

The unhealthy pig dataset was collected from 12 October to 19 October 2019, over the period from 00:00 to 24:00, at the Institute of Animal Health, Guangzhou Academy of Agricultural Sciences, Guangdong Province, China. A DS-2DC2204IW-D3 PTZ camera (Hikvision) was wall-mounted in the isolation pen to capture data from pigs exhibiting unhealthy behaviours. The camera operated at a sampling rate of 25 frames per second (25 fps) with an image resolution of 1920 × 1080 pixels. The pig breed in this dataset is specific-pathogen-free (SPF) Bama miniature pigs aged 40–50 days. Examples are shown in Figure 1c,d. Notably, all pigs in this dataset were infected with *Streptococcus suis* (*S*. *suis*), with clinical symptoms including unsteady standing, loss of appetite, and lethargy.

In this study, all procedures followed established animal welfare principles, and both housing environments were managed under the same husbandry protocols. Each pig was provided with 1.2 m^2^ pen space, and environmental conditions such as temperature and humidity were carefully regulated to ensure comfort. Adequate ventilation was also maintained to support the stocking density. The healthy pigs were housed on concrete flooring, whereas the unhealthy pigs were kept on plastic slatted floors to improve hygiene conditions. Some research has shown that pigs exhibit comparable behavioural patterns on concrete and slatted floors, indicating that flooring type has minimal influence on fundamental behaviours. Therefore, the difference in flooring does not affect the outcomes of this study. Detailed information of the healthy and unhealthy pig datasets is presented in Table 1.

#### 2.1.2. Data Processing and Annotation

In a large-scale pig farm, raw data inevitably contain erroneous data, such as segments with excessive noise or severe lighting interference. Therefore, it is essential to establish strict data exclusion criteria during the preprocessing stage to ensure the reliability and accuracy of subsequent analyses. In this study, we excluded data segments that were unusable due to equipment malfunction, as well as video clips with excessive noise or severe lighting interference.

In this study, we selected 28 videos from two datasets, including 18 videos of healthy pigs and 10 videos of unhealthy pigs. Among them, 9 videos of healthy pigs and 5 videos of unhealthy pigs were used as the test set. A total of 26 videos had a duration of 1 min (5 frames per second), including 300 frames. The 17th video clip from the healthy pigs’ and the 28th video clip from the unhealthy pigs’ datasets were used for the long-term (24 h) health assessment module.

**Figure 2:**
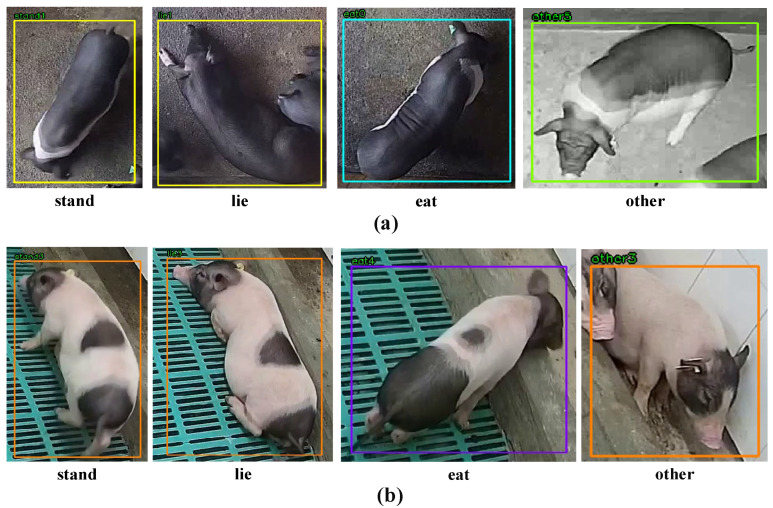
Examples of pig behaviour classification. (**a**) Examples of a healthy pig’s behavioural categories. (**b**) Examples of an unhealthy pig’s behavioural categories.

All videos were cropped using FFmpeg software (version 4.3) and manually annotated with DarkLabel to record positional information, behavioural categories, and identity (ID) information for each pig. It is noteworthy that animal behaviours are generally associated with meeting specific biological needs essential for the growth and survival of pigs and other species. These behavioural categories typically include ‘stand’, ’lie’, ‘eat’, and ‘other’ activities such as sniffing and semi-recumbent postures. Significant differences in the duration and frequency of these behaviours have been documented between healthy and unhealthy pigs, reflecting changes in their overall activity patterns [25]. The annotated behaviours are classified into four types: ‘stand’, ‘lie’, ‘eat’, and ‘other’. The definition of these four behaviours is described in Table 2, and the examples of behavioural classifications are illustrated in Figure 2.

### 2.2. Method

**Figure 3:**
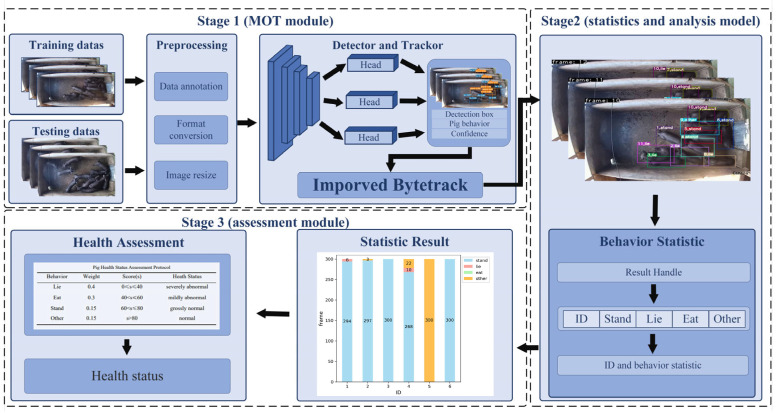
Pipeline for long-term behavioural analysis of pigs based on improved ByteTrack.

We propose the pigs’ health assessment framework based on multi-object behaviour tracking and analysis, including three main modules: an improved ByteTrack-based MOT module, a behaviour statistics and analysis module, and a health status assessment module. The framework is illustrated in Figure 3. Firstly, the YOLOX detector of the MOT module is employed to detect pigs to obtain their bounding boxes and confidence scores in each video frame. The detection results are then fed into an improved ByteTrack tracker, where tracking information (behaviour categories and IDs) is generated through data association and trajectory management strategies. Then, the behaviour statistics are used to collect the pigs’ ID numbers and behaviour category information to complete the behavioural analysis. Finally, the health status assessment module is applied to assess the pigs’ health status based on the time distribution of each behaviour derived from the statistical results.

#### 2.2.1. Improved ByteTrack

The original ByteTrack [26] exhibits two main limitations in pig behaviour tracking. First, the default detection box aspect ratios are not aligned with the elongated and dynamically varying body characteristics of pigs, which reduces the reliability of IoU-based data association. Second, in complex farm environments where severe occlusions frequently occur, the original ByteTrack tends to lose targets after short-term occlusion events, leading to a considerable number of IDs during tracking.

To address these issues, the improved ByteTrack introduces two key enhancements. The first is the rescaling of detection boxes, in which K-means clustering is used to derive aspect ratios that best fit pig body shapes. These optimised ratios are then applied to better align with pigs’ dynamic appearances and improve IoU association accuracy. The second enhancement is a trajectory interpolation post-processing strategy. By interpolating intermediate frames in which occlusions occur, this method effectively mitigates IDs in scenarios involving severe occlusion.

**Figure 4:**
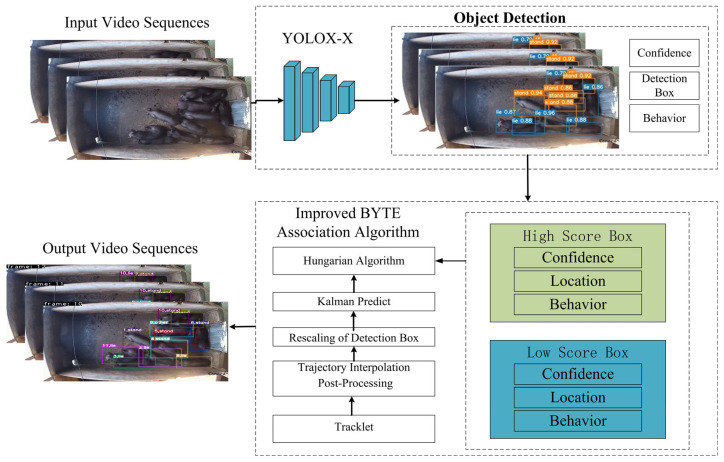
The flowchart of the improved ByteTrack.

The proposed improved ByteTrack includes two key stages: object detection and improved BYTE association algorithm. Its flowchart is shown in Figure 4. Firstly, in the detection stage, the YOLOX-X detection model is used to detect the input video frames. The model can accurately locate each pig and extract key information such as confidence, position, and category. Secondly, in the data association stage, the continuous trajectories of pigs are obtained using an improved BYTE data association algorithm, which combines Kalman filtering (KF) with the Hungarian algorithm. KF is used to predict the pigs’ future trajectories based on their motion dynamics, enhancing trajectory continuity and robustness. The Hungarian algorithm is then applied to optimally match these predicted trajectories with current trajectories by minimising the overall association cost, thereby effectively reducing IDs. Finally, the behaviour tracking results for each pig from consecutive video frames are obtained by the MOT tracker.

(1) Rescaling of Detection Box

To address the mismatch between the default detection box in the original ByteTrack and the body characteristics of pigs, we applied the K-means clustering algorithm to recalibrate the detection box based on pig-specific features. The cluster centres are iteratively updated during the K-means cluster centres calculated by Equation (1). The cluster centres are updated by Equation (2).

(1) $$d(x_{i},\mu_{j})=\sqrt{\sum_{k=1}^{n}{\left(x_{ik}-\mu_{jk}\right)}^{2}}$$

(2) $$\mu_{j}=\frac{1}{\left|C_{j}\right|}\sum_{x_{i}\in C_{j}}x_{i}$$

**Figure 5:**
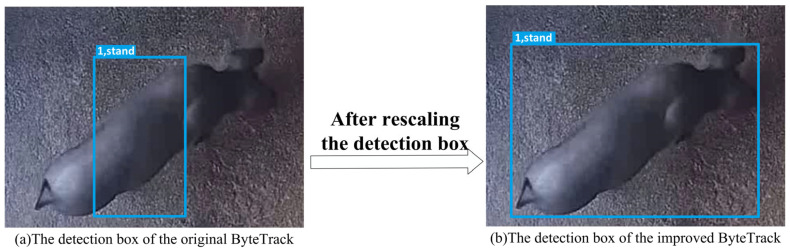
An example of the detection box between ByteTrack and the improved ByteTrack.

In Equation (1), $x_{i}$ represents the sample point of the dataset and $\mu_{j}$ denotes the centre of cluster j. $d(x_{i},\mu_{j})$ represents the distance between $x_{i}$ and $\mu_{j}$, and n denotes the feature number for each sample point. $x_{ik}$ and $\mu_{jk}$ are the k-th feature values of $x_{i}$ and $\mu_{j}$, respectively. In Equation (2), $\left|C_{j}\right|$ represents the number of sample points in the cluster j, and $\sum_{x_{i}\in C_{j}}$ denotes the summation over all sample points within cluster j. An example of the detection box is shown in Figure 5.

(2) Trajectory Interpolation Post-Processing Strategy

To avoid losing track of the target under the occlusion scene, we introduce a trajectory interpolation post-processing strategy based on BYTE data association. When a trajectory, T, is lost between two frames, $t_{1}$ and $t_{2}$, due to occlusion, and the current frame t satisfies $\left(t_{1}<t<t_{2}\right)$. The interpolation bounding box $B_{t}$ is calculated using Equation (3).

(3) $$B_{t}=B_{t_{1}}+(B_{t_{2}}-B_{t_{1}})\frac{t-t_{1}}{t_{2}-t_{1}}$$

where $B_{t}$, $B_{t1}$, and $B_{t2}$ correspond to the bounding box coordinates (top-left and bottom-right) at frames t, $t_{1}$, and $t_{2}$, respectively.

#### 2.2.2. The Behaviour Statistics and Analysis Module

**Figure 6:**
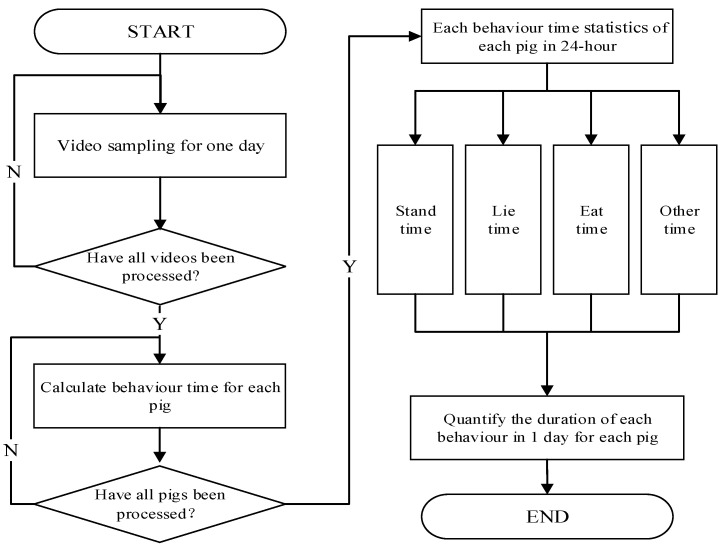
The overall pipeline of the behaviour statistics and analysis module.

The behaviour statistics and analysis module is used to collect the pigs’ ID numbers and behaviour category information to complete the pig behaviours analysis, which is illustrated in Figure 6. It includes three main stages.

(1) Video sampling. Video sequences are batch-sampled based on different time periods throughout a 24 h period. These videos are then processed using the improved ByteTrack to obtain the tracking results for behaviour tracking and analysis.
(2) Calculating pig behaviour times for each pig. We use the improved ByteTrack to process all the sampled videos to obtain tracking results. Then, the four behaviours’ (‘stand’, ‘lie’, ‘eat’, and ‘other’) durations of each pig in all videos are calculated according to our previous work [27].
(3) Quantify the duration of each behaviour. The time distribution of four behaviours in one day is quantified using the behaviour time of each pig from the sampled videos in 24 h.

#### 2.2.3. The Health Status Assessment Module

Healthy pigs exhibit a clear diurnal rhythm of activity [28,29], and they typically show increased activity following feeding, while spending approximately 70% of the day in an inactive state [30]. However, when pigs are in an unhealthy state, their behaviour undergoes marked changes, particularly in the allocation of time spent lying, eating, and standing. Previous research has reported that unhealthy pigs generally show reduced overall activity, prolonged lying duration, shortened eating time, and a lower frequency of standing behaviour [31,32]. In summary, we developed a health assessment module by integrating pig experts’ opinions with methods from previous studies [33,34]. The pig experts also evaluated the four pig behaviours by assigning health scores based on the time proportion of each behaviour. The assessment score tables for the ‘lie’, ‘eat’, ‘stand’, and ‘other’ behaviours are provided in Table 3, Table 4, Table 5 and Table 6, respectively. The ‘Proportion’ column in each table shows the percentage of each behaviour’s status within the total dataset.

A pig’s health status is assessed based on their overall health score according to Table 7, which is generated from the ‘lie’, ‘eat’, ‘stand’, and ‘other’ behaviour health scores (Table 3, Table 4, Table 5 and Table 6) for each pig. Therefore, the assessment tables and behaviour scoring weights are carefully designed by fully incorporating pig experts’ knowledge from pig farming and existing methods [1,14,22,25,30].

#### 2.2.4. Evaluation Metrics

In this study, we adopted three primary evaluation metrics: High-Order Tracking Accuracy (HOTA), Multiple Object Tracking Accuracy (MOTA), and Identification F1 Score (IDF1). HOTA provides a more comprehensive evaluation of overall performance by jointly considering both detection and association accuracy. MOTA assesses the effectiveness of object monitoring and trajectory maintenance, while IDF1 reflects the consistency and stability of identity assignment during tracking. Additionally, the study also employs three supplementary indicators—identity switches (IDs), False Positives (FP), and False Negatives (FN)—to further evaluate the robustness and reliability of the tracking performance.

#### 2.2.5. Experimental Platform and Environment

To evaluate the tracking performance of the improved ByteTrack and the accuracy of the health status assessment module, four experiments were conducted: (1) A pig tracking experiment to analyse the performance of the improved ByteTrack. (2) A comparison the improved ByteTrack with other mainstream MOT trackers. (3) A pig behaviour statistics experiment to calculate the duration of the four types of behaviour for each pig in the video. (4) A pig health assessment experiment to verify the accuracy of the health status assessment module. All experiments were carried out on a Linux platform running Ubuntu 18.04. The hardware configuration included a 12th Gen Intel(R) Core i9-12900KF CPU, an NVIDIA GeForce RTX 3090 GPU, and 32 GB of RAM. The software environment was based on PyTorch version 1.11.1, Python version 3.7, and CUDA version 11.3.

## 3. Results

### 3.1. Comparison of Improved ByteTrack’s MOT Results on Each Test Video

To validate the effectiveness of the improvements, we compared the performance of the improved ByteTrack with the original ByteTrack, as shown in Table 8. We observed that the improved ByteTrack demonstrated improvements in HOTA, IDF1, and IDs compared with ByteTrack. The average HOTA of the improved ByteTrack was 74.0%, reflecting a 1.3% increase compared to the original model. The average IDF1 was 89.4%, indicating a 3.1% improvement. The total number of IDs was 43, representing a 41.9% reduction compared to the ByteTrack.

As shown in Table 9, the improved ByteTrack achieved HOTA, MOTA, and IDF1 scores of 62.1%, 88.1%, and 76.0% on video 12, 65.7%, 88.5%, and 76.0% on video 13, and 54.9%, 77.5%, and 70.6% on video 18, respectively. For videos 14–17, the number of IDs was 0, indicating that the improved ByteTrack was able to track pigs more accurately in these videos without any identity switching.

The improved ByteTrack demonstrated improvements in HOTA, IDF1, and IDs. We attributed these enhancements to the following factors: the improved ByteTrack adjusts the bounding box proportions and enhances the HOTA and IDF1; using a trajectory interpolation post-processing strategy reduces the impact of occlusion, leading to a significant decrease in IDs. However, the class imbalance caused the model to favour learning high-frequency categories while neglecting low-frequency ones, which resulted in a decline in the MOTA. These results demonstrate that the overall performance of the improved ByteTrack is better and exhibits greater stability during object tracking compared with ByteTrack.

### 3.2. Comparison of Improved ByteTrack with Other MOT Methods

To evaluate the tracking performance of the improved ByteTrack, we compared it with JDE, DeepSORT, and TransTrack on the same dataset, and the results are summarised in Table 10. The improved ByteTrack achieved an HOTA of 74.0%, an MOTA of 92.2%, and an ID count of 43. Compared to JDE, DeepSORT, and TransTrack, its HOTA improved by 27.9%, 21.6%, and 17.7%, respectively, while MOTA increased by 38.9%, 7.3%, and 10.3%, respectively, and IDs decreased by 311, 410, and 207, respectively. Therefore, the improved ByteTrack algorithm demonstrates superior performance in the tracking tasks of group-housed pigs, confirming the effectiveness of the proposed improvements.

**Figure 7:**
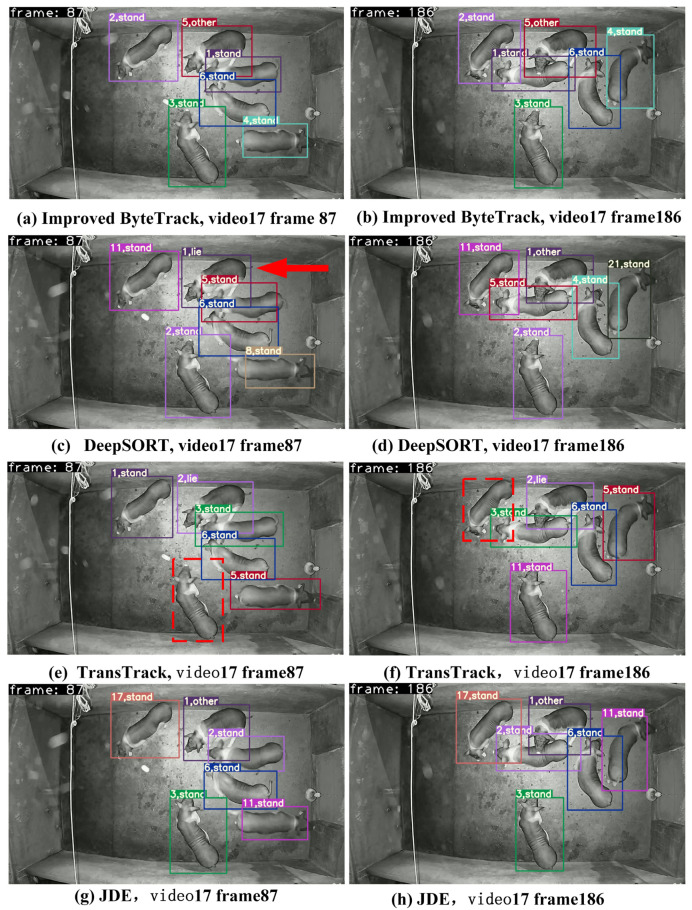
Comparison of partial tracking results between improved ByteTrack and other trackers.

The comparison of partial tracking results of the improved ByteTrack and other MOT algorithms is shown in Figure 7. The improved ByteTrack enabled stable and accurate pig tracking in frame 87 to 186 of video 17. For example, the behaviour of pigs with ID five and ID one in frame 87 of video 17 was the ‘other’ class. The improved ByteTrack and JDE can successfully predicted it as ‘other’ behaviour (Figure 7a,g), while DeepSORT and TransTrack incorrectly predicted it as “lie” behaviour (Figure 7c,e). In frame 186 of video 17, the improved ByteTrack assigned a maximum pig ID of six, matching the actual number of pigs (Figure 7b). The JDE, DeepSORT, and TransTrack method assigned maximum pig IDs of 17, 21, and 11, respectively (Figure 7h,d,f). TransTrack also exhibited pig target loss, as shown the red dashed box in Figure 7e,f. Overall, the improved ByteTrack exhibits more stable behaviour tracking and fewer IDs in large-scale pig farming.

### 3.3. The Statistical Analysis of Pig Behaviours

To accurately analyse and quantify pig behaviours, this study selected full-day videos of spotted pigs belonging to the breed officially named “MeiHua” and Bama miniature pigs from the healthy and the unhealthy pig datasets, respectively. The proposed improved ByteTrack was used for behaviour tracking. Additionally, a multi-timepoint batch sampling method was adopted for video sampling, which involved selecting multiple timepoints throughout the day and sampling at predefined intervals to ensure coverage of the entire 24 h period. Based on the sampled data, the times of the four pig behaviours for one day were obtained.

#### 3.3.1. The Short-Term Pig Behaviour Analysis

**Figure 8:**
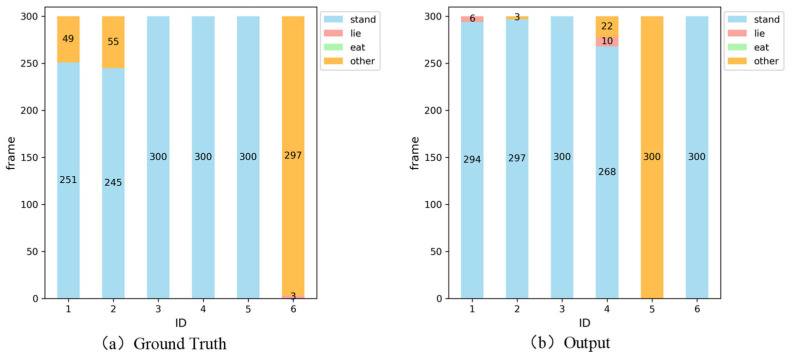
Bar chart of behavioural statistics from GT and model for video 17.

We conducted a short-term pig behavioural analysis (1 min, 300 frames from video 17). The behavioural statistics results of Ground Truth (GT) and the model are shown in Figure 8a,b. The horizontal coordinates represent the ID numbers of the pigs, and the vertical coordinates represent the number of frames for each behaviour.

**Figure 9:**
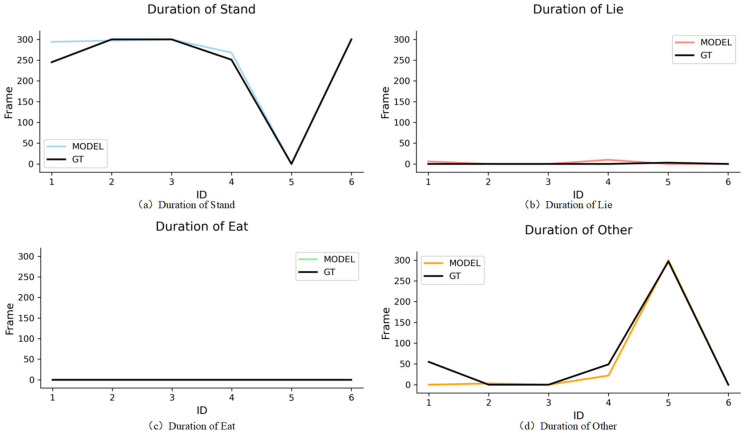
Frame distribution of the four behaviours from GT and model for video 17.

Due to potential discrepancies for the ID randomly assigned by the tracker differing from our annotated ID, we firstly established the correspondence between the IDs in GT and output IDs of the tracker according to the method of our previous work [27], as detailed in Table 11. Then, we constructed the line chart to record the duration distribution of the four behaviours from GT and the model for each pig in video 17, as shown in Figure 9. It can be found that the statistics results of the four behaviours can be obtained from a short-term video using our proposed tracker. Afterwards, we applied it to a long video (series3) for behavioural analysis.

#### 3.3.2. The Long-Term Pig Behaviour Analysis

**Figure 10:**
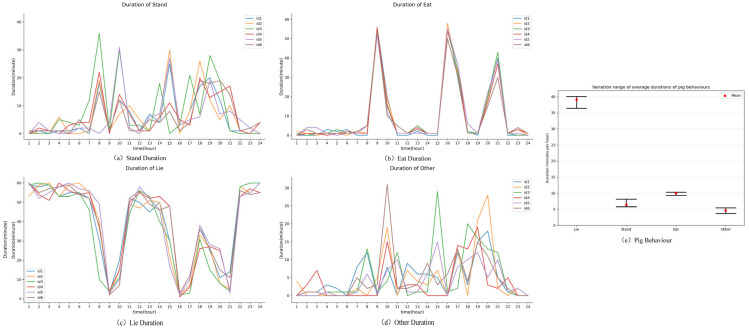
Long-term summary of the time distribution for each pig.

The long-term statistical results of the four behaviours from healthy pigs are shown in Figure 10. The horizontal coordinates represent the 24 h period, and the vertical coordinates represent the minutes of each behaviour for each hour. At three designated eat times (8:00 AM, 3:00 PM, and 7:40 PM), a significant peak in eat behaviour was observed (Figure 10b). This phenomenon may be attributed to the provision of fresh food, which stimulated ‘eat’ activity among the pigs. Due to the uneven distribution of food, pigs were required to forage actively. The average duration of the four pig behaviours, along with their respective fluctuation ranges, is presented in Figure 10e.

Approximately 1 to 2 h after feeding, a peak in lie behaviour was observed among the pigs (Figure 10c). The average lie behaviour duration reaches its highest level during nighttime. This trend may be attributed to the pigs’ need to rest after feeding in order to facilitate digestion, leading to increased ‘lie’ activity. Additionally, the circadian rhythm of the pigs may drive a higher frequency of lie behaviour during the night [25,35]. ‘Stand’ and ‘other’ behaviours increased noticeably around the three timepoints (Figure 10a–d). This increase may be due to the stimulation of appetite by the ‘eat’ activity, prompting pigs to stand more frequently and engage in interactions with other individuals.

From these statistical results in Figure 10e, it can be seen that the average duration of the pigs’ lie behaviour is 38.5 min per hour, with a variance of approximately 3.0; the average duration of standing behaviour is 6.5 min per hour, with a variance of approximately 1.5; the average duration of feeding behaviour is 10 min per hour, with a variance of approximately 1.0; and the average duration of other behaviour is 4.8 min per hour, with a variance of approximately 0.5. These results indicate that healthy pigs exhibit similar behavioural patterns across these daily activities.

The long-term behavioural statistics of unhealthy pigs are presented in Table 12. The results indicate that the unhealthy pigs spent most of their time lying down during daytime (from 12:00 PM to 6:00 PM). In the evening period (from 7:00 PM to 12:00 AM), there was a slight increase in ‘stand’ and ‘other’ behaviours. The unhealthy pigs predominantly remained in a lying posture in all day, and no ‘eat’ behaviour was observed. The reason may be attributed to symptoms caused by the *S. suis* infection, such as joint inflammation, loss of appetite, and lethargy, which reduce the pigs’ activity levels and result in prolonged ‘lie’ behaviour. The experiment conducted a 24 h behavioural monitoring session, and the results were highly consistent with manual observations, with only minimal deviations. These results indicate that long-term behaviour tracking demonstrates a high level of reliability [36].

### 3.4. The Results of the Pig Health Status Assessment

Based on the results of the pig behaviour statistics and analysis, we used the health status assessment module and obtained the proportions of time spent on the four behaviours for both healthy and unhealthy pigs, presented in Table 13 and Table 14, respectively.

For healthy six pigs, ‘lie’ behaviour accounts for 60.8–66.7%, ‘eat’ behaviour accounts for 15.8–17.5%, ‘stand’ behaviour accounts for 9.6–13.3%, and ‘other’ behaviour accounts for 6.3–9.2%. Specifically, a pig with ID3 exhibited a lower ‘lie’ proportion (60.8%) compared to other pigs, while a pig with ID6 had the lowest ‘eat’ proportion (15.8%). According to the assessment based on Table 13 and Table 14, all pigs were assessed as ‘healthy’.

In contrast, unhealthy pigs exhibited lie behaviour ranging from 94% to 99%, eat behaviour from 0% to 6.5%, stand behaviour from 0.8% to 3.7%, and ‘other’ behaviour from 0% to 2.9%. Compared with healthy pigs, unhealthy pigs spent significantly more time lying down and less time engaging in ‘eat’, ‘stand’, and ‘other’ activities.

The health assessment module (Section 2.2.3) was applied to the pigs listed in Table 14, and all pigs were evaluated as unhealthy. These assessment results are consistent with the actual observed health conditions of the pigs.

## 4. Discussion

The four behaviours (‘lie’, ‘eat’, ‘stand’, and ‘other’) are important indicators of a pig’s health status. Among these, lie behaviour has the greatest impact, as healthy pigs typically allocate a substantial portion of their daily time budget to lying. However, during illness, they further increase lying behaviour as a postural strategy to conserve the heat and metabolic energy required to cope with an infection [25,28,31,32,37,38]. In particular, infections such as streptococcal disease have been associated with solitary lying and reduced activity, which is consistent with the clinical signs observed in our unhealthy pigs [39]. Eat behaviour is the second most important, as the length of eat time may reflect their appetite and digestive health. Stand behaviour may be related to physical activity and energy levels, while ‘other’ behaviours may reflect psychological states. In summary, pig behaviours are closely linked to health status. However, few studies have analysed MOT results in depth to assess pig health.

With the development of pig behaviour tracking technologies, it has become increasingly feasible to perform health assessments based on tracking results. For example, Li et al. proposed a multi-behaviour detection method for group-housed pigs that integrates a YOLOX-based object detection module with an SCTS-SlowFast behaviour recognition module. This method effectively detects standing, lying, feeding, and walking behaviours [40]. In our previous work [27], we explored a YOLOv5-ByteTrack-based method for multi-object pig behaviour tracking, which accurately tracks and analyses pig behaviours over time. However, this work did not systematically assess pig health based on behaviour tracking and statistical analysis results.

To address this challenge, we propose a health assessment framework based on multi-object behaviour tracking and analysis. In this framework, the four primary behaviours are assigned specific weights to reflect their relative importance in evaluating pig health. The observed behaviours are then translated into corresponding scores to quantify the behaviours of pigs and assess their health status.

The health assessment results of this study show that, among the four behavioural categories observed within a 24 h period, healthy pigs spent on average 65.08% of their time lying, 16.68% eating, 10.62% standing, and 7.78% performing other behaviours. In contrast, pigs clinically diagnosed with a *Streptococcus suis* infection exhibited markedly different behavioural proportions, spending 95.10% of their time lying, 1% eating, 1.74% standing, and 1.66% on other behaviours. Evidently, the lying duration of unhealthy pigs increased significantly—by 30.02% compared with healthy pigs—whereas their eating time decreased sharply by 15.68%. These changes clearly indicate that unhealthy pigs typically display behavioural patterns characterised by prolonged lying, reduced feeding, and an overall decline in activity.

It is noteworthy that the typical clinical symptoms of an *S. suis* infection—such as arthritis, loss of appetite, and lethargy—substantially impair the mobility of pigs, making them more inclined to lie down for extended periods to minimise energy expenditure [39]. These clinical manifestations are highly consistent with the behavioural statistics observed in this study. Differences between healthy and unhealthy pigs are reflected not only in the proportional distribution of the four behavioural categories but also in their overall behavioural rhythms: healthy pigs show stable behaviour patterns with clear daily rhythms and normal fluctuations, whereas unhealthy pigs exhibit pathological behaviour characterised by reduced activity and diminished feed intake.

Although the present results successfully verify the feasibility of our approach, this study has certain limitations: (1) The behavioural data used for tracking were obtained through multi-timepoint video sampling, which may introduce a degree of error. (2) Our study was conducted on a dataset of limited size, especially regarding unhealthy pigs, due to the practical difficulties in data acquisition from unhealthy pigs. (3) Some factors such as management practices, feeding methods, and breed differences may have had some impact on the results. However, the aim of this work is to establish and validate a core analytical module and methodological framework for the automated monitoring and quantification of multiple behaviours to assess health status. The experimental data, which include lying, eating, standing, and other behaviours, were collected to validate the feasibility of our algorithmic modules and technical framework. These limitations do not affect the validity and feasibility of the algorithmic framework proposed in this paper and the alignment between our results and the actual data demonstrate the viability of the proposed method. In addition, our work is not an endpoint but a strategic first step. It establishes a cost-effective, scalable framework upon which a comprehensive multi-behaviour monitoring system (e.g., aggression and excretion patterns) can be built. For a deeper investigation into the behavioural characteristics of diseased pigs or a healthy diagnosis system, it is necessary to augment the dataset with pigs of the same breed, raised in identical environments, and under the same management. This will inform the direction of our future work.

## 5. Conclusions

In this study, we developed a pig health assessment framework based on multi-object behaviour tracking and analysis. The MOT model achieves an HOTA of 74.0%, an MOTA of 92.2%, an IDF1 of 89.4%, and recorded 43 IDs. Compared with the original ByteTrack, the improved version achieved a 1.3% increase in HOTA, a 3.1% improvement in IDF1, and a 41.9% reduction in IDs. Although the improved ByteTrack shows a 2.1% decrease in MOTA compared with the original ByteTrack, this slight reduction is acceptable given the substantial improvement in ID consistency resulting from the significant decrease in IDs. Additionally, using the proposed pig health evaluation framework, we assess each pig’s behavioural health based on the time distribution of four behaviours over a 24 h period. The results indicate that unhealthy pigs exhibited a 30.02% increase in the duration of lying behaviour compared to healthy pigs, which is consistent with the typical clinical manifestations of an *S. suis* infection [39]. These findings demonstrate the feasibility and effectiveness of the proposed health assessment framework based on behaviour statistics and analysis.

## Figures and Tables

**Table 1:** Detailed information of the healthy and unhealthy pig datasets.

Dataset	Split	Breed	Videos	Number of Pigs	Time
Healthy Pig	Train	Black Pig	1	11	1 min
			2	11	1 min
			3	10	1 min
			4	10	1 min
		MeiHua Pig	5	6	1 min
			6	6	1 min
			7	6	1 min
			8	6	1 min
			9	6	1 min
	Test	Black Pig	10	11	1 min
			11	11	1 min
			12	11	1 min
			13	11	1 min
		MeiHua Pig	14	6	1 min
			15	6	1 min
			16	6	1 min
			17	6	24 h
			18	6	1 min
Unhealthy Pig	Train	Bama mini-Pig	19	5	1 min
			20	5	1 min
			21	5	1 min
			22	5	1 min
			23	5	1 min
	Test	Bama mini-Pig	24	5	1 min
			25	5	1 min
			26	5	1 min
			27	3	1 min
			28	5	24 h

**Table 2:** Criteria for defining pig behavioural.

Definition of Pig Behaviour	Description of Behaviour	Example of Individual Pig Behaviour
stand	The pig maintains an upright posture with all four limbs fully supporting the body, without lying or sitting.	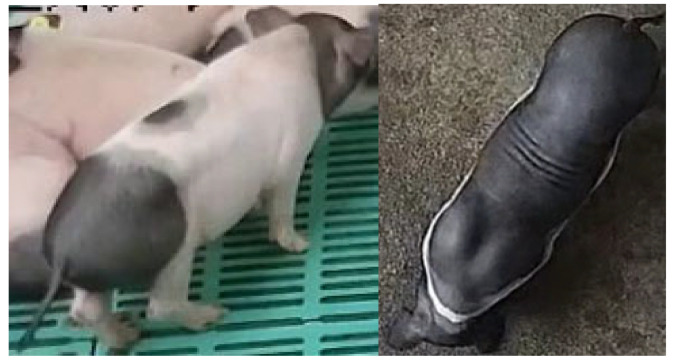
lie	The pig rests with the body in full contact with the ground in either lateral or sternal recumbency.	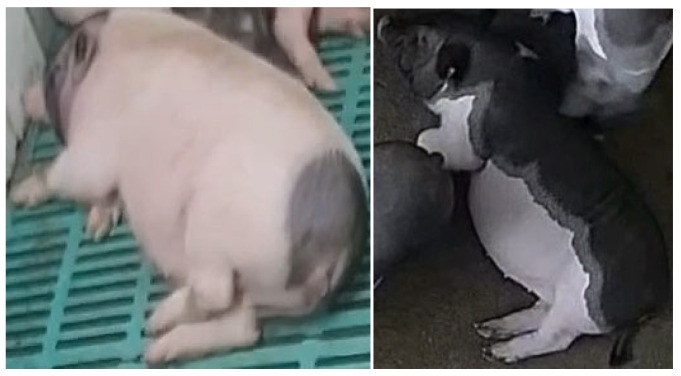
eat	The pig maintains full support of its body with all four limbs while the head is oriented downward toward the ground, engaging in ingestive or foraging actions.	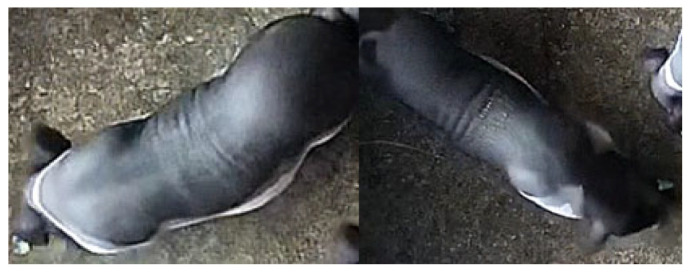
other	Behaviours that do not fall into the stand, lie, or eat categories, including, but not limited to, sniffing, semi-recumbent postures, and brief irregular or undirected activities.	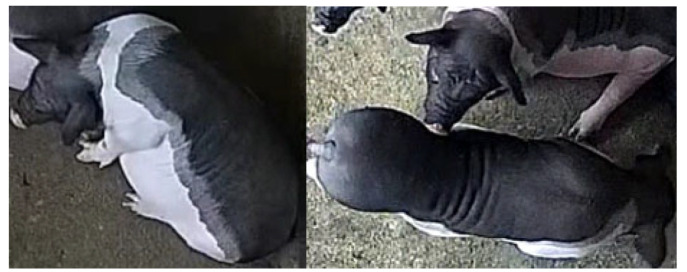

**Table 3:** Assessment table of pig ‘lie’ time.

Proportion	Status Assessment	Level	Score
0–20%	Almost no lying	Unhealthy	0
20–60%	Insufficient lying time	Possibly unhealthy	40
60–80%	Normal lying time	Healthy	100
80% and above	Excessive lying time	Possibly unhealthy	40

**Table 4:** Assessment table of pig ‘eat’ time.

Proportion	Status Assessment	Level	Score
0–5%	Insufficient eating time	Unhealthy	0
5–15%	Relatively insufficient eating time	Possibly unhealthy	40
15–20%	Normal eating time	Healthy	100
20–25%	Relatively prolonged eating time	Relatively healthy	70
25% and above	Excessive eating time	Unhealthy	0

**Table 5:** Assessment table of pig ‘stand’ time.

Proportion	Status Assessment	Level	Score
0–4%	Insufficient standing time	Unhealthy	0
4–8%	Relatively insufficient standing time	Possibly unhealthy	40
8–17%	Normal standing time	Healthy	100
17–25%	Relatively prolonged standing time	Relatively healthy	40
25% and above	Excessive standing time	Unhealthy	0

**Table 6:** Assessment table of pig ‘other’ behaviour time.

Proportion	Status Assessment	Level	Score
0–2%	Insufficient other time	Possibly unhealthy	40
2–4%	Relatively insufficient other behaviour time	Relatively healthy	70
4–15%	Normal other behaviour time	Healthy	100
15–20%	Relatively prolonged other behaviour time	Possibly unhealthy	40
20% and above	Excessive other behaviour time	Unhealthy	0

**Table 7:** Assessment table of the total health status.

Behaviour	Weight	Score(s)	Heath Status
lie	0.4	0 ≤ s ≤ 40	Severely unhealthy
eat	0.3	40 < s ≤ 60	Mildly unhealthy
stand	0.15	60 < s ≤ 80	Grossly healthy
other	0.15	s > 80	Healthy

**Table 8:** Tracking result of improved ByteTrack and ByteTrack.

M1.	HOTA/%↑	MOTA/%↑	IDF1/%↑	IDs↓	FP	FN
Improved ByteTrack	74.0	92.2	89.4	43	586	1095
ByteTrack	72.7	94.3	86.3	74	429	750

**Table 9:** Tracking results of improved ByteTrack for all test videos.

Video	HOTA/%↑	MOTA/%↑	IDF1/%↑	IDs↓	FP	FN
10	80.0	94.2	95.5	3	58	132
11	78.8	94.2	96.7	2	52	138
12	62.1	88.1	76.0	19	145	229
13	65.7	88.5	78.2	9	148	224
14	82.6	97.9	99.0	0	14	23
15	84.5	98.1	99.0	0	10	25
16	84.8	97.3	98.6	0	20	29
17	80.6	97.8	98.9	0	20	19
18	54.9	77.5	70.6	10	119	276
Total	74.0	92.2	89.4	43	586	1095

**Table 10:** Comparison of tracking results from the improved ByteTrack and other trackers.

MODEL	HOTA/%↑	MOTA/%↑	IDF1/%↑	IDs↓	FP	FN
JDE	46.1	53.3	55.0	354	6990	3031
DeepSORT	52.4	84.9	58.1	453	1371	1527
TransTrack	56.3	81.9	70.7	250	850	2917
Improved ByteTrack	**74** **.** **0**	**92** **.** **2**	**89** **.** **4**	**43**	**586**	**1095**

**Table 11:** Correspondence between GT IDs and output IDs of tracker.

ID value in GT	2	3	4	1	6	5
ID value in Tracker	1	2	3	4	5	6

**Table 12:** The long-term statistics results of unhealthy pigs.

Behaviour	ID	Time												
		12	13	14	15	16	17	18	19	20	21	22	23	24
Lie	1	49.7	60	60	60	60	60	60	54.6	60	45.6	59.7	60	50.5
	2	50	60	60	60	60	60	60	60	60	60	59.5	60	50.7
	3	30.7	60	60	60	60	60	60	45.2	60	31	60	40	50
	4	23.2	60	60	60	55.5	60	60	60	60	60	50	60	60
	5	44.6	60	60	60	60	60	60	59.1	60	60	52.1	60	60
Stand	1	0	0	0	0	0	0	0	0	0	10	0	0	0
	2	0	0	0	0	0	0	0	0	0	0	0	0	9.3
	3	11	0	0	0	0	0	0	0.1	0	17.6	0	0	0
	4	4	0	0	0	0	0	0	0	0	0	10	0	0
	5	5	0	0	0	0	0	0	0.9	0	0	0	0	0
Eat	1	0	0	0	0	0	0	0	0	0	0	0	0	0.3
	2	0	0	0	0	0	0	0	0	0	0	0	0	0
	3	7.6	0	0	0	0	0	0	11.2	0	1.3	0	20	10
	4	1.2	0	0	0	4.1	0	0	0	0	0	0	0	0
	5	0	0	0	0	0	0	0	0	0	0	0	0	0
Other	1	0.3	0	0	0	0	0	0	5.4	0	4.4	0.3	0	9.2
	2	0	0	0	0	0	0	0	0	0	0	0.3	0	0
	3	0.7	0	0	0	0	0	0	3.6	0	10.1	0	0	0
	4	21.7	0	0	0	0.3	0	0	0	0	0	0	0	0
	5	0.4	0	0	0	0	0	0	0	0	0	7.9	0	0

**Table 13:** Assessment results of healthy pig.

ID	Lie	Eat	Stand	Other	Status
1	65.4%	16.7%	9.6%	7.9%	Healthy
2	64.6%	16.7%	10.4%	8.3%	Healthy
3	60.8%	16.7%	13.3%	9.2%	Healthy
4	66.7%	16.7%	10.8%	6.3%	Healthy
5	66.3%	17.5%	10.0%	6.7%	Healthy
6	66.7%	15.8%	9.6%	8.3%	Healthy
Total	65.08%	16.68%	10.62%	7.78%	

**Table 14:** Assessment results of unhealthy pig.

ID	Lie	Eat	Stand	Other	Status
1	96.1%	0%	1.2%	2.5%	Unhealthy
2	98.7%	0%	1.2%	0%	Unhealthy
3	87.9%	6.5%	3.7%	1.9%	Unhealthy
4	94.6%	0.7%	1.8%	2.9%	Unhealthy
5	98.2%	0%	0.8%	1.0%	Unhealthy
Total	95.10%	1%	1.74%	1.66%	

## Data Availability

Data will be made available on request.
